# Endoscopic sphincterotomy and endoscopic biliary stenting do not affect the sensitivity of transpapillary forceps biopsy for the diagnosis of bile duct adenocarcinoma

**DOI:** 10.1186/s12876-022-02402-x

**Published:** 2022-07-05

**Authors:** Toshinori Aoki, Eizaburo Ohno, Takuya Ishikawa, Yasuyuki Mizutani, Tadashi Iida, Kentaro Yamao, Takeshi Yamamura, Kazuhiro Furukawa, Masanao Nakamura, Takashi Honda, Masatoshi Ishigami, Hiroshi Yatsuya, Hiroki Kawashima

**Affiliations:** 1 Department of Gastroenterology and Hepatology, Nagoya University Graduate School of Medicine, 65 Tsuruma-cho, Showa-ku, Nagoya, 466-8550 Japan; 2 Department of Endoscopy, Nagoya University Hospital, Nagoya, Japan; 3 Department of Public Health and Health Systems, Nagoya University Graduate School of Medicine, Nagoya, Japan

**Keywords:** Klatskin tumor, Biopsy, Endoscopic sphincterotomy, Stent, Endoscopic retrograde cholangiopancreatography

## Abstract

**Background:**

The pathological evaluation of tissues with cholangitis is considered difficult, which can often occur after endoscopic sphincterotomy (EST) and endoscopic biliary stenting (EBS). This study aimed to evaluate the influence of a history of EST and EBS on the sensitivity of transpapillary forceps bile duct biopsy (TB) for bile duct adenocarcinoma.

**Methods:**

This retrospective study included consecutive cases of bile duct adenocarcinoma in which TB was performed before July 2020 until the number exceeded that required to support statistical and noninferiority analyses of the sensitivity of TB between patients with and without each variable. The incidence of postprocedural adverse events related to each factor was also investigated.

**Results:**

Overall, 280 samples were required in each group, and 437 subjects (792 samples) were included. The sensitivity of TB was 63.6% for the subjects and 59.6% for the biopsy samples. For the biopsy samples, the sensitivity did not differ significantly between samples from patients with and without a history of EST (59.1% vs. 58.9%, *P* = 0.952) and EBS (62.1% vs. 55.4%, *P* = 0.065). The sensitivity was significantly higher for samples from patients with jaundice (67.9% vs. 57.0%, *P* = 0.008). There were significantly fewer procedure-related adverse events in patients with a history of EST (10.8% vs. 19.0%, *P* = 0.017) and EBS (12.0% vs. 21.7%, *P* = 0.005).

**Conclusions:**

A history of EST or EBS did not influence sensitivity of TB but significantly decreased the incidence of adverse events. To safely and reliably perform TB to diagnose bile duct adenocarcinoma, planning, including for EST and EBS, is necessary.

**Supplementary Information:**

The online version contains supplementary material available at 10.1186/s12876-022-02402-x.

## Background

Bile duct adenocarcinoma is difficult to diagnose through imaging in many cases, even with contemporary imaging methods [1–4], and a definite diagnosis requires pathological examination using transpapillary bile duct forceps biopsy (TB), brush cytology, or bile juice cytology performed at the same time as endoscopic retrograde cholangiopancreatography (ERCP). In principle, biopsy permits the collection of a larger amount of tissue than brush or bile juice cytology, providing more information for the diagnosis of bile duct adenocarcinoma. Thus, the diagnostic sensitivity of biopsy is higher than that of brush cytology, but the sensitivity has varied from 30 to 88% among reports, and the results are unsatisfactory [5]. And sample collection can be technically difficult and may be a risk factor for post-ERCP pancreatitis [6].

Many patients who undergo TB have previously undergone endoscopic sphincterotomy (EST) or endoscopic biliary stenting (EBS) for jaundice and cholangitis. EST is a risk factor for cholangitis in the long term [7, 8], and EBS can cause bacterial contamination in the bile duct [9]. The pathological evaluation of tissues with epithelial cholangitis is considered difficult, but the influence of a history of EST and EBS on the sensitivity of bile duct biopsy for pathological diagnosis has not been examined. In contrast, the sensitivity of TB has been reported to increase in the presence of jaundice [10].

Based on this background, the primary aim of this study was to examine whether a history of EST, EBS, and jaundice affects the sensitivity of TB for obtaining a histopathological diagnosis of bile duct adenocarcinoma. The secondary aim was to evaluate the relationship between these factors and procedure-related adverse events after ERCP.

## Methods

### Study design

This is an observational study comparing the sensitivity of TB diagnosis in later-confirmed bile duct adenocarcinoma patients between with and without certain procedures or conditions. The study retrospectively collected data necessary for the analyses from the clinical records of Nagoya University Hospital through July 2020. The study was performed after obtaining approval from Institutional Review Board of Nagoya University Hospital (approval number 2016–0032) and performed according to the guidelines described in the Helsinki Declaration for biomedical research involving human patients (Clinical trial registration number: UMIN000025631; date of registration: 11/01/2017).

### Hypothetical noninferiority trial design and the sample size calculation

We hypothesized that sensitivity of TB performed after certain procedures or conditions (i.e., histories of EST, EBS, or jaundice) was noninferior to that done without such procedures. Therefore, the minimum number of samples necessary to demonstrate the absence of a significant difference (noninferiority) in the sensitivity of TB for obtaining a pathological diagnosis in the presence and absence of each factor was first calculated. Then, consecutive patients with a final diagnosis of extrahepatic bile duct adenocarcinoma who underwent TB in July 2020 or earlier at Nagoya University Hospital were retrospectively surveyed until a sufficient number of samples was identified.

### Intervention

**Fig. 1:**
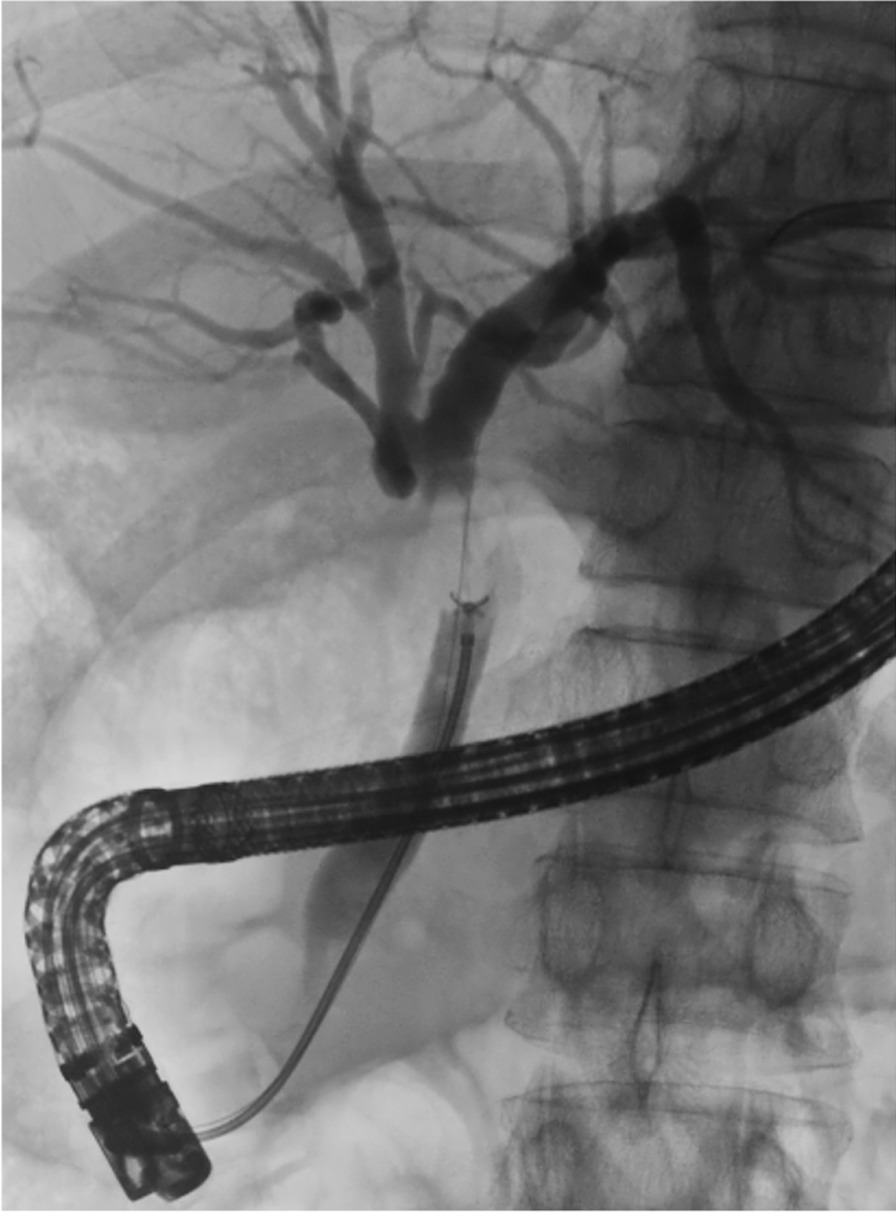
Samples were obtained by TB from bile duct adenocarcinoma

ERCP was performed by multiple endoscopists who had performed ≥ 200 ERCP procedures. One or more samples were obtained by TB from a bile duct lesion during ERCP (Fig. 1), and all samples were numbered according to the order of collection. ERCP and biopsy were performed with a duodenoscope (JF-260 V, TJF-260 V, Olympus Medical, Tokyo, Japan) and forceps (Radial Jaw 4P, Boston Scientific), respectively. Two experienced pathologists diagnosed each sample histopathologically as adenocarcinoma, suspected adenocarcinoma, atypical tissue, no malignancy, or insufficient material. If the diagnosis differed between the two pathologists, a consensus was reached by discussion. If the histopathological diagnosis differed from the clinical findings, ERCP was repeated as necessary to perform transpapillary biopsy.

### Definitions

Cases in which EST was performed in a session prior to TB were defined as having a history of EST. Macroscopic type (papillary/nodular or flat) of bile duct adenocarcinoma was differentiated by intraductal ultrasonography during ERCP. Regarding the histopathological diagnoses based on biopsy specimens, adenocarcinoma and suspected adenocarcinoma were defined as positive findings, and other diagnoses were defined as negative findings. The final diagnosis was made based on the histopathological diagnosis made from samples acquired during surgery and evaluations performed during a 6-month or longer clinical course, including imaging techniques such as computed tomography (CT) and magnetic resonance imaging (MRI). Jaundice was defined as a serum total bilirubin level ≥ 3 mg/dL measured immediately before ERCP. Procedure-related adverse events were graded based on the guidelines of the American Society for Gastrointestinal Endoscopy [11]. Bleeding was defined as clinical evidence of bleeding within 7 days after the procedure and a decrease in Hb ≥ 2 g/dL. The consensus definition and classification of procedure-related pancreatitis from Cotton et al. [12] was used. Cholangitis was diagnosed according to the 2018 Tokyo guidelines [13], and post-ERCP cholangitis was defined as new developments after ERCP.

### Statistical analysis

The necessary number of samples was determined using SAS noninf macro with the following specifications based on past reports: sensitivity of biopsy at 60%, the α error at 5%, power at 80%, and the noninferiority margin at 10%. The analysis was performed by SAS 9.4. χ2 tests were used for statistical comparisons, with *P* < 0.05 indicating a significant difference and *P* ≥ 0.05 indicating noninferiority. These calculations were performed using SPSS Statistics ver. 27.

## Results

The analysis yielded 280 samples as the size necessary in each group. Accordingly, we obtained data from a total of 472 ERCP procedures performed in 437 patients (792 samples) with a final diagnosis of extrahepatic bile duct adenocarcinoma who underwent TB between January 2012 and July 2020. In 335 patients, the diagnosis was histopathologically made from specimens obtained at surgery. The clinical characteristics of 437 patients included in the present study were as follows: median age: 72 years (25–90 years); sex: 309 males and 128 females; tumor location: perihilar in 349 patients (631 samples) and distal in 90 patients (161 samples) (including overlapping cases); number of biopsies performed: 1 in 215 cases, 2 in 161 cases, 3 in 32 cases, and ≥ 4 in 29 cases; and macroscopic type: papillary/nodular in 199 patients (342 samples), flat in 236 patients (447 samples), and nontypable in 2 patients (3 samples).

Of the 437 patients, 172 (308 samples) had a history of EST, 253 (462 samples) had no history of EST (naïve papilla). All 172 cases had undergone EST at a previous institution, and none of them had undergone EST at Nagoya University Hospital. 12 had a history of endoscopic papillary balloon dilatation and were excluded from the EST analysis. In the 472 ERCP sessions, an endoscopic biliary stent had been placed in 275 (475 samples) and not placed in 189 (303 samples). Only 7Fr or 8.5Fr plastic stents were used. Eight patients who had EBS prolapse or underwent percutaneous transhepatic biliary drainage (PTBD) placement were excluded from the EBS analysis. Of the 275 ERCP sessions with EBS, 152 were ERCP sessions with a history of EST. The median total bilirubin level was 1.3 mg/dL (range: 0.1–33.1 mg/dL). The bilirubin level was not measured in 2 cases, and these cases were excluded from the analysis of the effect of jaundice (Table 1).

**Table 1:** Clinical characteristics

Variable		Value
Age (median)	Years (range)	72 (25–90)
Sex	Male Female	309 cases 128 cases
Tumor localization^a^	Perihilar Distal	349 cases (631 samples) 90 cases (161 samples)
Number of biopsies	1 2 3 ≥ 4	215 cases 161 cases 32 cases 29 cases
Macroscopic type^b^	Papillary/nodular Flat	199 cases (342 samples) 236 cases (447 samples)
Medical history of EST	Yes No (untreated papilla)	172 cases (308 samples) 253 cases (462 samples)
EBS^c^	Yes No	275 times (475 samples) 189 times (303 samples)
Median total bilirubin level	mg/dL (range)	1.3 (0.1–33.1)

*EST* endoscopic sphincterotomy, *EBS* endoscopic biliary stenting

^a^Including overlapping cases, ^b^2 cases were nontypable, ^c^Number of ERCP procedures. Patients who had EBS prolapse or underwent PTBD placement were excluded

The sensitivity of TB was 63.6% for the patients (positive in 278 patients) and 59.6% for the biopsy samples (positive in 472 samples) (Table 2). There were no cases of false-positive histopathological diagnoses based on biopsy specimens. For the biopsy samples, the sensitivity of TB was noninferior for samples from patients with a history of EST compared to for those without this history (59.1% vs. 58.9%, *P* = 0.952) or for samples with EBS compared to for those without EBS (62.1% vs. 55.4%, *P* = 0.065). The sensitivity of TB was significantly higher for samples from patients with jaundice (190 samples) than for without jaundice (589 samples) (67.9% vs. 57.0%, *P* = 0.008); in distal cases than in perihilar cases (71.4% vs. 56.6%, *P* = 0.001); and in papillary/nodular type tumors than in flat-type tumors (68.4% vs. 47.7%, *P* < 0.001). The sensitivity of TB tended to be higher in patients without cholangitis at the time of ERCP than in those with cholangitis, although the difference was not significant (Table 3).

**Table 2:** Sensitivity of bile duct biopsy

	Sensitivity of bile duct biopsy
Patients	63.6% (278/437 cases)
Biopsy samples	59.6% (472/792 samples)

**Table 3:** Univariate analysis of the sensitivity of bile duct biopsy

Factor		Sensitivity of bile duct biopsy	*P*-value
Tumor localization	Perihilar Distal	56.6% (357/631) 71.4% (115/161)	0.001
Macroscopic type	Papillary/nodular Flat	68.4% (234/342) 47.7% (213/447)	< 0.001
History of EST	Yes No (untreated papilla)	59.1% (182/308) 58.9% (272/462)	0.952
EBS	Yes No	62.1% (295/475) 55.4% (168/303)	0.065
Jaundice ^a^	Yes No	67.9% (129/190) 57.0% (336/589)	0.008
Cholangitis ^b^	Yes No	51.8% (42/81) 60.4% (429/710)	0.136

*EST* endoscopic sphincterotomy, *EBS* endoscopic biliary stenting

^a^Total bilirubin level ≥ 3.0 mg/dL. Cases without data were excluded, ^b^Cholangitis was diagnosed according to the Tokyo Guidelines 2018

Pancreatitis, cholangitis, and cholecystitis were observed as ERCP-related adverse events. The overall incidence of procedure-related adverse events was significantly lower in patients with a history of EST and EBS than in those without these histories (EST: 10.8% vs. 19.0%, *P* = 0.017; EBS 12.0% vs. 21.7%, *P* = 0.005). In particular, the incidence of pancreatitis was significantly lower in the patients with a history of EST or EBS. In contrast, post-ERCP cholangitis was more frequent in patients with EBS. The presence of jaundice had no significant effect on the incidence of adverse events (Tables 4, 5, 6).

**Table 4:** Univariate analysis of ERCP-related adverse events in patients with and without a history of EST, calculated by ERCP sessions

Adverse events		History of EST	No history of EST (naïve papilla)	*P-*value
All adverse events		10.8% (20/185)	19.0% (52/273)	0.017
Pancreatitis	Mild Moderate Severe	0.5% (1/185) 0.5% (1/185) –	9.5% (26/273) 1.8% (5/273) 0.7% (2/273)	< 0.001
Cholangitis		9.7% (18/185)	6.2% (17/273)	0.166
Cholecystitis		–	0.4% (1/273)	0.410

*EST* endoscopic sphincterotomy

**Table 5:** Univariate analysis of ERCP-related adverse events in patients with and without EBS, calculated by ERCP sessions

Adverse events		With EBS^a^	Without EBS	*P*-value
All adverse events		12.0% (33/275)	21.7% (41/189)	0.005
Pancreatitis	Mild Moderate Severe	0.4% (1/275) 0.4% (1/275) –	13.8% (26/189) 2.6% (5/189) 1.1% (2/189)	< 0.001
Cholangitis		10.9% (30/275)	3.7% (7/189)	0.005
Cholecystitis		0.4% (1/275)	–	0.407

*EBS* endoscopic biliary stenting

^a^Patients who had EBS prolapse and underwent PTBD placement were excluded

**Table 6:** Univariate analysis of ERCP-related adverse events in patients with and without jaundice, calculated by ERCP sessions

Adverse events		With jaundice^a^	Without jaundice	*P*-value
All adverse events		16.1% (19/118)	16.0% (56/349)	0.989
Pancreatitis	Mild Moderate Severe	7.6% (9/118) 1.7% (2/118) 0.8% (1/118)	5.4% (19/349) 1.1% (4/349) 0.3% (1/349)	0.646
Cholangitis		5.1% (6/118)	8.9% (31/349)	0.187
Cholecystitis		0.8% (1/118)	–	0.085

^a^Jaundice was defined as a total bilirubin level ≥ 3.0 mg/dL. Cases without data were excluded

## Discussion

It is important to acquire histological evidence prior to performing highly invasive surgery [14–16] and administering anticancer drug treatment for bile duct adenocarcinoma. This data collection process has been examined in studies on sample collection numbers [17] and improving diagnostic performance by immunostaining [18]. The sensitivity of biopsy for bile duct cancer has varied among reports, but the sensitivity of 63.6% in the current study is not markedly different from the 59% found by de Bellis et al. [19] and 75.6% obtained by Yamamoto et al. [20].

Many factors may influence the sensitivity of TB. The sensitivity is higher for distal bile duct cancer than for perihilar bile duct cancer [20]. The reasons may be that the diameter and stricture of the hilar bile duct are narrower than those of the distal bile duct, making it difficult for forceps to spread sufficiently, and the distance from the papilla to the tumor is longer, making tissue collection difficult. As other factors, Naito et al. [10] reported that the sensitivity of TB was higher in patients with a total bilirubin ≥ 4 mg/dL than for those with a bilirubin level < 4 mg/dL, and Nishikawa et al. [21] found that TB had a significantly higher sensitivity for macroscopic nonflat (papillary/nodular)-type tumors than for flat-type tumors. Nonetheless, the current study is the first to examine the effects of a history of EST and EBS on the diagnostic sensitivity of TB. EST is likely to cause duodenum reflux and bacterial infection of bile [7, 8], and EBS can similarly cause infection of bile with intestinal bacteria due to reflux from the duodenum [9, 22]. Both are considered to be risk factors for cholangitis. Since our previous study shows EST can cause cholangitis in ENBD cases [23], TB is basically performed without EST at Nagoya University Hospital. In this study, there were no cases in which EST was performed at Nagoya University Hospital. It is generally considered that inflammation-induced atypical changes in the bile duct epithelium increase the difficulty of obtaining a histopathological diagnosis, but there is limited evidence for this view. In the current study, the sensitivity of TB for reaching a diagnosis was demonstrated to be noninferior in patients with a history of EST and EBS, which are predicted to induce inflammation of the bile duct and reduce the diagnostic sensitivity. In addition, biopsy sensitivity tended to be higher in cases with cholangitis in this study. The reason for this is unknown, but we suggest that one of the reasons is that many cholangitis cases underwent EST or EBS, which allows for better handling of the forceps and collection of larger tissue. The sensitivity of TB was significantly higher in patients with distal bile duct cancer, jaundice (total bilirubin ≥ 3 mg/dL) and papillary/nodular type cancer, as previously reported. The sensitivity of TB was not significantly different in the presence of cholangitis at the time of ERCP, but the sample size may have been small.

The incidences of all adverse events and post-ERCP pancreatitis were significantly lower in patients with a history of EST or EBS. Multiple cannulation procedures into the bile duct and pancreatography have been reported as risk factors for post-ERCP pancreatitis [24], which suggests that the lower incidence of post-ERCP pancreatitis in patients with a history of EST or EBS was due to the ease of inserting the forceps into the bile duct, helping to avoid risk factors. In contrast, the incidence of post-ERCP cholangitis was significantly higher in patients who had undergone EBS. Therefore, we believe that TB should be performed after EST, especially in cases with a high risk of post-ERCP pancreatitis, such as those in which cannulation of the bile duct is difficult. Patients with a history of EST or EBS were divided into groups with a history of EST only, EBS only, or both, and the incidence of all adverse events and post-ERCP pancreatitis in each group was significantly lower than those in the group without both. On the other hand, the two groups with EBS tended to have a higher incidence of cholangitis than the group without both, respectively. The comparison of adverse events in the four groups is shown in Additional file 1: Table S1, Additional file 2: Table S2, Additional file 3: Table S3.

The limitations of this study include its retrospective single-center design and the lack of an investigation on specificity. However, we believe that our research will be useful in planning examination and treatment for patients with bile duct adenocarcinoma.

## Conclusions

We found that the sensitivity of TB in obtaining the pathological diagnosis for bile duct adenocarcinoma was not reduced in patients who had undergone EST or EBS, and these patients also had a significantly reduced incidence of adverse events. Thus, to safely and reliably perform TB for the diagnosis of bile duct adenocarcinoma, planning, including for EST and EBS, is necessary.

## Supplementary Information

**Additional file1**. **Table S1**: ERCP-related adverse events in patients with a history of EST and no EBS and those with neither a history of EST nor EBS, calculated by ERCP sessions

**Additional file2**. **Table S2**: ERCP-related adverse events in patients without a history of EST and with EBS and those with neither a history of EST nor EBS, calculated by ERCP sessions

**Additional file3**. **Table S3**: ERCP-related adverse events in patients with a history of EST and EBS and those with neither a history of EST nor EBS, calculated by ERCP sessions

## Data Availability

The datasets used and analyzed in the current study are available from the corresponding author on reasonable request.

## Abbreviations

CT: Computed tomography
EBS: Endoscopic biliary stenting
ERCP: Endoscopic retrograde cholangiopancreatography
EST: Endoscopic sphincterotomy
MRI: Magnetic resonance imaging
PTBD: Percutaneous transhepatic biliary drainage
TB: Transpapillary bile duct forceps biopsy
