# Supplementary material for: Endoscopic sphincterotomy and endoscopic biliary stenting do not affect the sensitivity of transpapillary forceps biopsy for the diagnosis of bile duct adenocarcinoma
Source: BMC Gastroenterol. 2022 Jul 5;22:329. doi: 10.1186/s12876-022-02402-x (PMC9258154; doi:10.1186/s12876-022-02402-x)
Supplement: Supplementary file 2 — Additional file2. Table S2: ERCP-related adverse events in patients without a history of EST and with EBS and those with neither a history of EST nor EBS, calculated by ERCP sessions [file 12876_2022_2402_MOESM2_ESM.docx]

Supplementary Table 2. ERCP-related adverse events in patients without a history of EST and with EBS and those with neither a history of EST nor EBS, calculated by ERCP sessions

| Adverse events | | No History of EST / With EBS | No history of EST / Without EBS | *P*-value |
| --- | --- | --- | --- | --- |
| All adverse event | | 10.5% (12/114) | 25.7% (39/152) | 0.002 |
| Pancreatitis | Mild  Moderate  Severe | 0 % (0/114)  0 % (0/114)  0 % (0/114) | 16.4% (25/152)  3.3% (5/152)  1.3% (2/152) | <0.001 |
| Cholangitis | | 9.6% (11/114) | 3.9% (6/152) | 0.060 |

EST, endoscopic sphincterotomy

EBS, endoscopic biliary stenting
